# Sarcopenia in a patient with most serious complications after highly invasive surgeries treated with nutrition, rehabilitation, and pharmacotherapy: a case report

**DOI:** 10.1186/s40780-021-00197-9

**Published:** 2021-04-06

**Authors:** Michiyo Tatsumi, Satomi Kumagai, Takahiro Abe, Soichi Murakami, Hiroshi Takeda, Toshiaki Shichinohe, Yuko Watanabe, Shinsuke Katayama, Shiaki Hirai, Aiko Honda, Yoh Takekuma, Mitsuru Sugawara

**Affiliations:** 1 Department of Pharmacy, Hokkaido University Hospital, Kita 14, Nishi 5, Kita-Ku, Sapporo, Hokkaido 060-8648 Japan; 2 Department of Nutrition, Hokkaido University Hospital, Sapporo, Hokkaido Japan; 3 Department of Rehabilitation, Hokkaido University Hospital, Sapporo, Hokkaido Japan; 4 Department of Gastroenterological Surgery II, Hokkaido University Hospital, Sapporo, Hokkaido Japan; 5 Pathophysiology and Therapeutics, Faculty of Pharmaceutical Sciences, Hokkaido University, Sapporo, Hokkaido Japan

**Keywords:** Sarcopenia, Postoperative, Nutrition therapy, Exercise, Pharmacotherapy, Total parenteral nutrition, Branched-chain amino acids (BCAA), Rehabilitation

## Abstract

**Background:**

Several studies have reported the implementation of nutrition therapy and rehabilitation for acute and critical illnesses. However, rehabilitation nutrition for elderly sarcopenia patients with extremely severe postoperative complications during hospitalization has not yet been established.

**Case presentation:**

We report the case of a 70-year-old man with sarcopenia that developed as a postoperative complication of the surgical resection of perihilar cholangiocarcinoma and left the patient bedridden from prolonged malnutrition and muscle weakness. The patient’s general condition improved after a nearly 6-month intervention by our Nutrition Support Team (NST) that combined nutrition, exercise, and pharmacotherapy.

**Conclusions:**

The appropriate timing and order of pharmacotherapy, nutrient administration, exercise therapy, and team collaboration may enable elderly patients with severe (secondary) sarcopenia and postoperative complications to regain self-sustained walking.

## Background

Elderly patients are at an increased risk of sarcopenia and postoperative complications after highly invasive surgeries [1]. In addition, they are prone to muscle strength loss due to inactivity [2] and malnourishment during hospitalization [3]. Surgery for perihilar cholangiocarcinoma is a particularly invasive type of gastrointestinal surgery with a high rate of perioperative complications. The morbidity rates after surgery are high because it is more likely to present added potential risk factors, such as obstructive jaundice with or without cholangitis [4] [5]. Malnutrition is associated with adverse perioperative outcomes [6]. Patients recovering from severe illnesses may experience significant muscle mass loss. In a previous randomized controlled trial, Jones et al. [7] reported that enhanced physiotherapy, structured exercise, and amino acid supplementation (including glutamine) may aid physical recovery in elderly patients recovering from a prolonged period of illness. Patients with intensive care unit stay of 5 days or more were recruited in those study. However, participants were excluded if they had renal failure (requiring regular dialysis). Hegerova et al. [8] also reported that early nutritional supplements and early physiotherapy preserve muscle mass and independence in elderly patients hospitalized for acute disease. However, lean body mass (LBM) progression was only − 0.4 kg during the 6-month period in this study group. The factors contributing to both trial outcomes, physical recovery, and muscle mass remain unclear. We report the case of an elderly bedridden patient with secondary sarcopenia who developed postoperative complications including acute kidney injury (AKI) (requiring dialysis) and hepatic failure. The patient’s LBM and general condition greatly improved after a 6-month intervention by Nutrition Support Team (NST). We will discuss some potential factors contributing to favorable outcomes in our case.

## Case presentation

### Patient

The patient was a 70-year-old man with a history of cerebral infarction (no paralysis), hypertension, and duodenal ulcers; he had no history of allergies. On admission, he was independently ambulatory. The Eastern Cooperative Oncology Group Performance Status (ECOG PS; range, 0–4; with higher scores indicating worse disability) was 0 [9]. Serum transferrin, pre-albumin, and retinol-binding protein levels were 237 mg/dL (range: 190–300), 23.4 mg/dL (range: 22–40), and 1.90 mg/dL (range: 3.6–4.2), respectively, before the operation. He underwent resection of the right and caudate lobes of the liver, extrahepatic bile duct resection, choledochojejunostomy, and jejunostomy for perihilar cholangiocarcinoma.

### Need for intervention

During the first 3 months after the operation, the patient experienced hepatic failure, AKI, disseminated intravascular coagulation (DIC), and pneumonia. Hemodialysis was performed three times per week. The patient was bedridden for the entire day, and his ECOG PS was 3–4. The enteral tube was placed in the jejunum to administer medicine and nutrition; however, enteral nutrition was stopped repeatedly owing to the worsening of postoperative complications or gastrointestinal symptoms. Consequently, he received both parenteral and enteral nutrition with a total energy of 1200 kcal/day and 25.0 g/day protein. Physical findings and serum levels related to renal function are shown in Table 1. When his body composition was analyzed using the InBody® (Inbody S20; Biospace, Seoul, Korea) system, the skeletal muscle mass, skeletal muscle mass index (SMI), fat mass, body fat, and extracellular water (ECW) to total body water (TBW) ratio were 14.2 kg, 5.85 kg/m^2^, 19.4 kg, 39.0%, and 0.436, respectively. The patient had edema, ascites, and a pleural effusion. His dry weight estimated by subtracting the excess fluid from the actual body weight was 47.0 kg; the patient lost 10.0 kg in about 5 months. He met the diagnostic criteria for sarcopenia by the Asian Working Group for Sarcopenia (AWGS) [10] and was classified as severely malnourished. He underwent repeated cycles of constipation and diarrhea.

**Table 1:** Physical findings and serum levels related to renal function

Level	Day −126	day1	day112	day200
Height (cm)	155	155	155	155
Weight (kg)	57.1	49.8	47.8	55.9
Body mass index (kg/m^2^)	23.8	20.7	19.9	23.3
Serum creatinine (mg/dL)	0.80	2.36	1.06	0.77
eGFR creatinine (mL/min)	72.6	22.2	53.3	75.4
Serum cystatin C (mg/dL)	0.95	3.47	2.94	2.81
eGFR cystatin (mL/min)	74.1	13.9	18.0	19.1

Day −126: first day of hospitalization

Day 1: first day of nutritional intervention

### Intervention goals

The target energy intake was set at 35.0 kcal/kg/day (1700 kcal/day). The target protein intake was 1.20 g/kg/day (60.0 g/day) and 0.80 g/kg/day (40.0 g/day) when on and off dialysis, respectively, based on the consensus among the NST, the nephrologist, and the gastrointestinal surgeon, and consistent with the 2016 Clinical Practice Guideline for Acute Kidney Injury in Japan [11] and recommendation of the third revision (2013) of the guidelines for parenteral and enteral nutrition in Japan [12]. The use of intravenous- and enteral-nutrition routes was evaluated continuously. The patient was assessed as having AKI which gradually improved but transitioned from acute to chronic kidney disease. The nephrologist assessed the need for weekly hemodialysis, which the patient received as needed. Priority was given to patient mobilization, balance of energy intake and expenditure, prevention of complications associated with bed rest, and prevention of the progression of generalized deconditioning.

### Clinical course

The patient was fed via a combination of total parenteral nutrition, peripheral parenteral nutrition, and enteral nutrition; blood-glucose levels were monitored. We evaluated the patient’s physical findings, renal function test values (Table 1) and adjusted laxative prescriptions to improve bowel movements. Dialysis was stopped on day 56. On day 112 of the NST intervention, the protein dose was 1.40 g/kg/day, and the energy and protein doses were stabilized. The intake of essential nutrients was sufficient with continuous enteral nutrition alone at a slow rate throughout the day (Table 2).

**Table 2:** Composition of the administered solutions

Composition	Aminoleban®EN	Renalen®MP	ENEVO®	Total amount/ day
Dose (mL)	400	500	250	1150
Administration time	9:00–17:00	17:00–3:00	3:00–9:00	24 h
Administration rate (mL/h)	50	50	42	–
Protein (g)	27.0	28.0	13.5	68.5
Carbohydrate (g)	63.0	128	39.6	230.6
Lipids (g)	7.40	22.4	9.60	39.4
Na (mEq)	3.40	20.9	10.0	34.3
Isoleucine (g)	3.85	1.44	0.72	5.99
Leucine (g)	4.07	2.64	1.35	8.06
Valine (g)	3.20	1.84	0.87	5.91

ENEVO® (250 mL/300 kcal) and Renalen®MP (250 mL/400 kcal) are liquid nutritional supplements. Aminoleban®EN (50 g/213 kcal) is a powdered nutritional supplement that is dissolved in 200 mL water. ENEVO® is a polymeric formula consisting of a milk-protein isolate, whey-protein concentrate, and soy protein isolate. Aminoleban® consists 50% of gelatin hydrolysate, to which 8 amino acids have been added. Renalen®MP is a polymeric formula consisting of milk protein

The patient’s limbs were strong enough to perform activities of daily living (ADL) in bed, so upper body muscle training was started 5 days a week for 20 min. The aim of this training was to extend the sitting time on the bed and to acquire ADL-movement ability. Moreover, lower-limb muscle training was initiated to prevent the loss of skeletal muscle. As the patient’s nutritional status improved, the proportion of strength training in the regimen was gradually increased. The patient was able to stand unassisted on day 100 of the intervention; hence, walking training was started.

Renalen®MP was administered via an enteral route as an energy-dense nutrition formula to meet the target energy goals. Furthermore, we added branched-chain amino acids (BCAA)-enriched nutritional supplements (i.e., ENEVO® and Aminoleban®EN) because they are expected to promote protein synthesis [13]. Initially, Renalen®MP was administered during the daytime, and the patient frequently complained of gastroesophageal reflux and bloating during tube feeding, probably because of the high fat content in Renalen®MP. Because of these complaints, it was difficult for him to continue rehabilitation. Therefore, we changed the time of administration of Renalen®MP from daytime to nighttime (Table 2). The patient was able to walk independently on day 150. He could also ingest a small amount of food orally. On day 200, the skeletal-muscle mass, SMI, fat mass, body fat, and ECW/TBW ratio were 19.1 kg, 7.95 kg/m^2^, 17.6 kg, 31.4%, and 0.436, respectively. The ECOG PS was 2. The rehabilitation was continued, so that the patient could be discharged.

### Outcomes

**Fig. 1:**
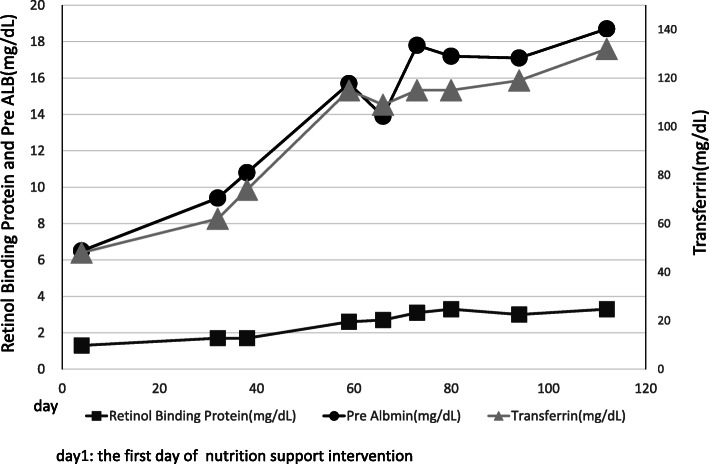
Serum levels of retinol binding protein (mg/dL), Pre albumin (mg/dL), and transferrin (mg/dL) from Day 1 to Day 112

**Fig. 2:**
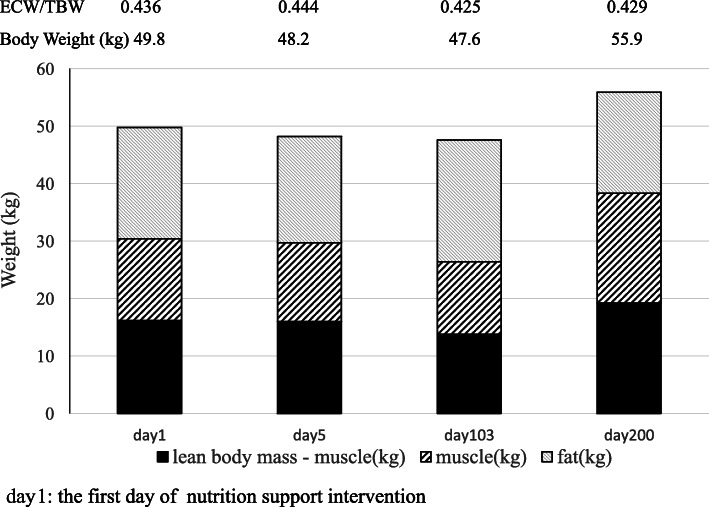
Body weight and muscle-fat analysis measured by InBody® on days 1, 5, 103, and 200

**Fig. 3:**
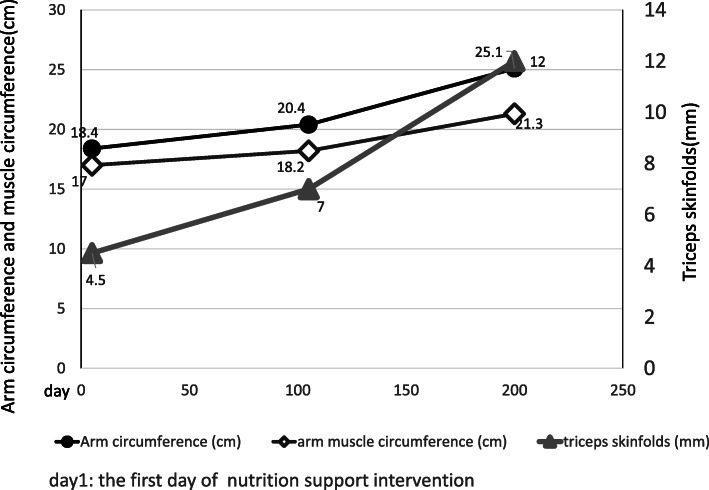
Brachial muscle analysis. The arm circumference (standard value: 26.8 cm), arm muscle circumference (standard value: 23.6 cm), and triceps skin folds (standard value: 10.0 mm) on days 5, 105, and 200. The standard value is based by the Japanese Anthropometric Reference Data 2001

The patient was able to receive daily nutrition both enterally and orally. The dynamic nutritional-assessment parameters and brachial muscle analysis increased over time (Figs. 1, 3). Body weight and LBM slightly decreased by day 103 but increased after day 103. On day 200, they have improved to what it was almost before the surgery (Fig. 2). He continued rehabilitation because his bowel movements were controlled.

## Discussion and conclusions

During severe illness and comorbidity, skeletal muscle and function are lost. Muscle mass is determined by the balance between protein synthesis and breakdown. Exercise and BCAA (particularly leucine) increase protein synthesis by upregulating the anabolic pathway that promotes intramuscular protein synthesis [14]. Kim et al. found that exercise and amino acid supplementation together may be effective in enhancing not only muscle strength but also combined variables of muscle mass, walking speed, and strength in sarcopenic women [15]. In our patient, despite the initiation of physical training immediately after the surgery, muscle mass did not improve until nutrition intervention, as he had a lack of nutrition. This finding was in accordance with those of previous studies [15, 16]. At the time of muscle protein catabolism, the physiotherapist tried to maintain ADL and muscle strength without increasing the exercise load until the required energy was administered. Therefore, the activity level was intentionally set to the sitting position. The patient performed strength training mainly while sitting to maintain physical function, but not for muscle hypertrophy. Moreover, the training encouraged voluntary ADL. Strength training required for each movement was performed continuously since the intervention. As the patient’s nutritional status improved, we gradually increased the proportion of strength training in the regimen. On day 100, he was able to stand unassisted; therefore, we initiated walking training. He was able to walk from his bedroom to a nurse’s station and back independently by day 150.

Jones et al. [7] did not report the total energy, total protein intake, findings, or muscle strength. Regarding the outcome data, a systematic review judged a high risk of bias due to a lack of power and follow-up for over 20% of the participants [17]. In the study by Hagerova et al. [8], participants were excluded if they had weight loss recently. The length of hospital stay for all patients was 11 ± 7 days, and nutritional intake was tolerated during hospitalization. LBM did not change markedly in the intervention group. Two RCTs [7, 8] were premised on restarting oral intake after admission [17]. In our patient, LBM improved from 29.7 kg to 38.3 kg. It was considered that appropriate and continuous training as well as nutrition focused on catabolism and anabolism enabled improvement of his physical strength.

Liquids that contain fats are reportedly emptied from the stomach more slowly than liquids that do not contain fats because the presence of fat causes secretion of cholecystokinin [18]. In contrast, dietary proteins with any amino-acid composition are processed by the stomach at the same speed regardless of whether they are amino acids, peptides, or polypeptides [19]. Aminoleban®EN is lower in fat (0.925 g per hour) than Renalen®MP (2.4 g per hour) and ENEVO® (1.6 g per hour). His gastrointestinal symptoms diminished enough to enable the continuation of rehabilitation after the order of administration time of nutrition was changed as Table 2.

Our patient experienced complications such as multiple organ failure, including an AKI meeting the Kidney Disease: Improving Global (KDIGO) diagnostic criteria [20], DIC, and chronic kidney disease; further, he had been repeatedly admitted to the intensive care units. Before the intervention by NST, the patient showed persistently suppressed adaptive immunity and low-grade inflammation indicating accelerated protein catabolism [21]. In Japan, it was used as a standard practice to restrict protein intake in patients with renal impairment regardless of the severity of the condition. However, this changed with the publication of the Clinical Practice Guideline for AKI in Japan [11]. Clinical Practice Guideline for AKI [11] suggest the target energy intake and dose of protein depend on the severity and identity of the underlying disease. NST, the nephrologist, and the gastrointestinal surgeons decided that the first goal should be to control inflammation and dialysis-induced protein catabolism. Therefore, the target protein intake was set as 1.20 g/kg/day (60.0 g/day) and 0.80 g/kg/day (40.0 g/day) when on and off dialysis, respectively. The nephrologist assessed the need for weekly dialysis and gastrointestinal surgeons evaluated nutritional prescriptions according to the patient’s symptoms and test results every 3 days or 1 week until the dialysis was completely stopped, and inflammation reduced.

The effect of exercise on nutritional status and body composition has been reported previously [22]. Nutritional rehabilitation in critically ill patients on and after intensive care units is difficult [23]. Recovery from moderate to severe AKI is heterogeneous and can take up to several months [24]. A previous study reported that LBM was significantly low in hemodialysis patients, and hemodialysis increased protein breakdown [25]. Therefore, it is important to recover from dialysis for effective nutrition. In our case, a pharmacist monitored the prescribed medications to prevent renal injury. A registered dietitian and pharmacist checked the protein intake and renal function every week until dialysis was stopped. At the time of anabolism, protein intake gradually increased to 1.40 g/kg/day.

Three points should be considered in this case: First, the InBody®-measurement results were reference values because the impedance was reversed. The ECW/TBW was over 0.4 suggesting ascites retention due to postoperative liver failure and fluid retention due to AKI. Therefore, the InBody® measurement may not be accurate. Body weight and LBM gradually decreased by day 103 (Fig. 2). However, the arm circumference, arm muscle circumference, and triceps skin folds increased over time (Fig. 3). This seems to be due to recovered renal function from improved postoperative complications, and the edema and ascites were reduced. Second, the Asian Working Group for Sarcopenia requires a decrease in the SMI and grip strength or walking speed to define sarcopenia. In this case, the grip strength and walking speed were measured before the operation but could not be measured after the operation. Third, the measurement of protein catabolism biomarkers was not performed. Therefore, we evaluated the time of anabolism when serum concentrations of C-reactive proteins were below 4 mg/dL [26].

Our patient with prolonged malnutrition due to postoperative complications was treated by initiating a 6-month-long NST intervention that combined nutrition, exercise therapy, and pharmacotherapy. The results demonstrated that the appropriate timing of nutritional counseling, exercise therapy, and team collaboration can enable elderly patients with severe (secondary) sarcopenia due to postoperative complications to recover and regain self-sustained walking.

## Data Availability

Data sharing does not apply to this article as no datasets were generated or analyzed during the current study.

## Abbreviations

ADL: Activities of daily living
AKI: Acute kidney injury
BCAA: Branched-chain amino acids
DIC: Disseminated intravascular coagulation
ECOG PS: Eastern Cooperative Oncology Group performance status
ECW: Extracellular water
LBM: Lean body mass
NST: Nutrition support team
SMI: Skeletal muscle mass index
TBW: Total body water
